# Combined Calorimetry, Thermo-Mechanical Analysis and Tensile Test on Welded EN AW-6082 Joints

**DOI:** 10.3390/ma11081396

**Published:** 2018-08-09

**Authors:** Philipp Wiechmann, Hannes Panwitt, Horst Heyer, Michael Reich, Manuela Sander, Olaf Kessler

**Affiliations:** 1 Institute of Materials Science, Faculty of Mechanical Engineering and Marine Technology, University of Rostock, Albert Einstein-Str. 2, 18059 Rostock, Germany; philipp.wiechmann@uni-rostock.de (P.W.); olaf.kessler@uni-rostock.de (O.K.); 2 Institute of Structural Mechanics, Faculty of Mechanical Engineering and Marine Technology, University of Rostock, Albert Einstein-Str. 2, 18059 Rostock, Germany; hannes.panwitt2@uni-rostock.de (H.P.); horst.heyer@uni-rostock.de (H.H.); manuela.sander@uni-rostock.de (M.S.); 3 Competence Centre CALOR, Department Life, Light & Matter, Faculty of Interdisciplinary Research, University of Rostock, Albert-Einstein-Str. 25, 18059 Rostock, Germany

**Keywords:** AlMgSi alloy, EN AW-6082, welding, mechanical properties, microstructure, DSC, thermo-mechanical analysis, digital image correlation, tensile test, numerical simulation

## Abstract

Wide softening zones are typical for welded joints of age hardened aluminium alloys. In this study, the microstructure evolution and distribution of mechanical properties resulting from welding processes of the aluminium alloy EN AW-6082 (AlSi1MgMn) was analysed by both in-situ and ex-situ investigations. The in-situ thermal analyses included differential scanning calorimetry (DSC), which was used to characterise the dissolution and precipitation behaviour in the heat affected zone (HAZ) of welded joints. Thermo-mechanical analysis (TMA) by means of compression tests was used to determine the mechanical properties of various states of the microstructure after the welding heat input. The necessary temperature–time courses in the HAZ for these methods were measured using thermocouples during welding. Additionally, ex-situ tensile tests were done both on specimens from the fusion zone and on welded joints, and their in-depth analysis with digital image correlation (DIC) accompanied by finite element simulations serve for the description of flow curves in different areas of the weld. The combination of these methods and the discussion of their results make an essential contribution to understand the influence of welding heat on the material properties, particularly on the softening behaviour. Furthermore, the distributed strength characteristic of the welded connections is required for an applicable estimation of the load-bearing capacity of welded aluminium structures by numerical methods.

## 1. Introduction

Wrought EN AW-6082 (AlSi1MgMn) alloy, as an age hardening aluminium alloy, has excellent weldability, corrosion resistance and mechanical strength and is widely used in the automobile and shipbuilding industries. The major alloying elements of this aluminium alloy 6082 are Mg and Si, which can increase the strength of the alloy through precipitation hardening. The welding of aluminium alloys can lead to defects such as porosity, incomplete fusion and hot cracking, and thus the welding work can be challenging. Age hardening aluminium alloys such as 6082, whose strength is increased by precipitation hardening, always exhibit phase transformation and a softening phenomenon because of the heat input generated during the welding process [1,2]. A proven method for investigations of such softening is the characterisation of microstructure and mechanical properties of welded joints by metallography as well as by standard load tests. Results (e.g., [3]) show the decreases of base material strength within the heat affected zone due to the dissolution of strengthening precipitates. For deeper knowledge and understanding of the softening phenomena, an in-situ characterisation of the microstructure development would be preferable. Differential scanning calorimetry is a suitable technique to record the precipitation and dissolution behaviour in situ during the heat treatment of aluminium alloys [4]. The method was initially developed for analysis of the precipitation behaviour during cooling after solution annealing and was subsequently expanded to the analysis of the short-term heat treatment of age-hardening aluminium alloys [5,6]. For a correct understanding of softening phenomena within the HAZ, knowledge of phase transformations during heating would be necessary. In this work, DSC was used for the first time to investigate the dissolution and precipitation behaviour of an age-hardened AlMgSi alloy when heated under typical temperature–time curves of a welding process. The results of the thermal analysis are discussed alongside the distributed mechanical properties of the HAZ, which have been determined in two ways. First, welded joints were investigated with elaborate load tests supported by numerical analysis. Second, the mechanical properties of a wide variety of microstructures caused by welding heat input were determined through thermo-mechanical analysis. The results of this work contribute to a better understanding of the development of mechanical properties in HAZ and make it possible to provide realistic material models for structure–mechanical investigations using the finite element method. In particular, the aim of the present project was to use the obtained results for the representation of the material characteristics of welded aluminium cross joints and to predict their limit load behaviour with numerical simulations.

## 2. Materials and Methods

### 2.1. Investigated Aluminium Alloy 

The experiments of this study were performed on a wrought aluminium alloy, EN AW-6082 (BIKAR-Aluminium GmbH, Korbußen, Germany), which was supplied as a 10 mm thick plate in the initial state T651. According to DIN EN 515 the treatment T651 includes solution annealing, quenching, stretching by 1.5% to 3% and subsequent artificial aging. EN AW-4047 (MTC GmbH, Meerbusch, Germany) was used as welding filler material for welding specimens. The chemical composition of EN AW-6082 and fusion zone material of a butt joint determined with optical emissions spectroscopy (OES) is given in Table 1 in addition to the specifications from DIN EN 573-3 [7].

Table 2 shows the mechanical properties in three different directions (rolling direction 0°, 45° and 90°, as in [8]) of the base material determined from tensile tests. A comparison with the standard shows that the properties of the base material fit or exceed the required values in all directions. The differences between the directions in the present rolled plate material are negligible compared to the differences in extruded material (e.g., Chen et al. [9]). Thus, isotropic behaviour can be assumed [10]. In this study, the mechanical properties from the 0°-specimens are used for the base material.

Furthermore, for different methods investigating the material behaviour several different samples were used. Table 3 gives an overview of the different specimen geometries and dimensions.

The high strengths in aluminium alloys are achieved in particular by precipitation hardening [12]. The precipitation sequence of Al-Mg-Si alloys was described by Edward and Dutta et al. [13,14]. An overview of these precipitates with information on dimensions, coherence, shape and further remarks was given by Polmear [15]. In Al-Mg-Si alloys, the beta phase results in maximum strengths [13]. 

The precipitation behaviour of several Al-Mg-Si alloys during cooling was investigated with DSC and microstructure analysis (optical microscopy (OM), SEM and TEM) [16,17,18,19]. Two different reaction areas, high (HTR) and low temperature reactions (NTR), were detected. In part, there is also a third middle temperature reaction (MTR). The high temperature reactions were correlated with the precipitation of Mg_2_Si and the low temperature reactions of the precipitation of precursor phases. Precipitation behaviour depend strongly on initial state and chemical composition The critical cooling rate of 6082 can vary by factor of 10 depending on Mg and Si content [20]. 

In [21], the precipitation behaviour of the same batch of 6082 in the same initial state as in this study was analysed depending on different annealing conditions. The precipitation behaviour depends above all on whether there is a complete or incomplete dissolution of secondary particles at the onset of cooling.

The dissolutions and precipitations of Al-Mg-Si alloys during heating were also analysed with DSC and it was linked to the mechanical properties by TMA [5,21,22]. Osten et al. [21] investigated the dissolution and precipitation behaviour of several Al-Mg-Si alloys, including 6082, in various initial states during heating and has assigned the measured peaks to specific reactions through extensive literature research.

### 2.2. Welding Procedure and Temperature Measurements

**Figure 1:**
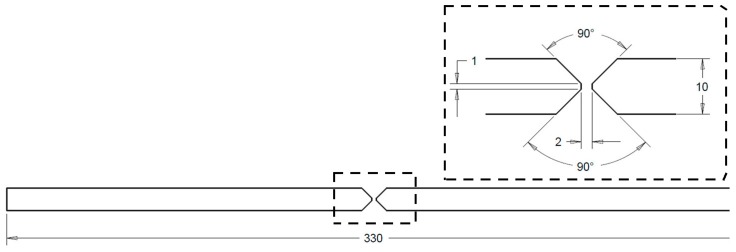
Prepared plates for butt welding, length of plates was 500 mm, lengths in mm.

**Figure 2:**
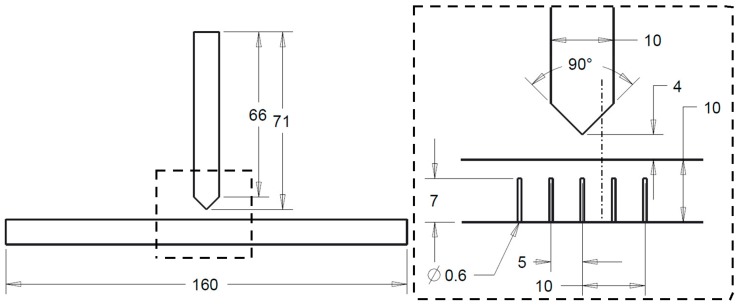
Sketch of prepared T-joint including drill holes for thermocouples, lengths in mm.

Considering the aim of the project, butt welded joints and T-joints were used (see Figure 1 and Figure 2), which were processed manually with metal inert gas welding (MIG) with three and four beads, respectively. Plates of EN AW-6082 T651 were welded with EN AW-4047 (wire diameter 1.2 mm) as weld filler material. Welding was conducted with direct current and positive polarity. A mixture of argon and helium (70%/30%) was applied as shielding gas. A ceramic weld pool backing was used for all joints. Further welding parameters are listed in Table 4.

A temperature–time course in the heat-affected zone during a real welding process is needed as input data for differential scanning calorimetry and for thermo-mechanical analysis. Eight thermocouples (Type K, 0.5 mm, Therma Thermofühler GmbH, Lindlar, Germany) that were completely inserted in drilled holes simultaneously measured the temperature with a frequency of 50 Hz. The geometry of the prepared aluminium sheets is displayed in Figure 2 including the positions of the holes for thermocouples. The diameter of the holes was 0.6 mm, slightly larger than the diameter of thermocouple wire, to ensure that the thermocouples could be positioned at the end of drilled holes. The length of this T-joint was 240 mm and the thermocouple holes were drilled lengthwise at 80 and 160 mm from the edge.

### 2.3. Differential Scanning Calorimetry

The heating rate range of 0.01–5 K s^−1^ was investigated by direct DSC with two types of calorimeters: CALVET-type heat-flux DSC (DSC 121 and Sensys, Setaram, Caluire-et-Cuire, France) for slower (0.01–0.1 K s^−1^) and power-compensated DSC for faster (0.3–5 K s^−1^) scanning rates (Pyris Diamond and Pyris DSC 8500, PerkinElmer, Waltham, MA, USA). The samples used for heat-flux DSC had a cylindrical geometry with 6 mm diameter, 21.65 mm height and a mass of 1600 mg. Cylindrical samples with 6.4 mm diameter, 1 mm height and a mass of 80 mg were investigated in the power-compensated DSC devices. All experiments were carried out with an alloyed sample in one micro furnace and a pure–aluminium reference (99.9995% purity) with the same geometry in the other micro furnace. The samples and references were packed in pure-aluminium crucibles.

For investigation of very fast heating rates, which are typical for the HAZ during welding, direct DSC cannot be used, because the heating rate limit of the devices is exceeded. Instead, the indirect DSC method was used. Zohrabyan et al. [23] developed the differential reheating method to extend the temperature rate range. The schematic procedure of this method is shown in Chapter 3.2 together with its results. Rapid heating took place in the quenching dilatometer Bähr 805 A/D (BÄHR Thermoanalyse GmbH, Hüllhorst, Germany). The device is explained in Chapter 2.4. For indirect DSC, the samples had the same geometry (diameter of 6.4 mm, height of 1 mm, mass of 80 mg) as for direct DSC in the power-compensated devices. The samples were heated with rates from 20 to 100 K s^−1^ to temperatures of 200 °C to 450 °C, respectively, with an interval of 25 K. To preserve the state of the material at the maximum temperature, the samples were immediately quenched with maximum gas flow from He. After heat treatment, the samples were directly frozen at −80 °C until being reheated in the DSC device.

Reheating in the DSC device was performed with a scanning rate of 1 K s^−1^ to a maximum temperature of 575 °C. 

The data processing of raw measured heat flow curves applied in this study was described in detail by Fröck et al. [24]. To obtain high-quality DSC results, the following sequence of experiments was conducted: sample measurement–baseline measurement, sample measurement. This is an efficient method to obtain a baseline for each sample measurement immediately. Baseline measurements were carried out with two pure aluminium references in the micro furnaces and the same temperature program as for the sample measurements. Baseline measurements were made to ascertain the current device specific curvature, which can change significantly within hours. This curvature is removed by subtracting the baseline determined in a timely manner.

The comparison of DSC curves of different sample masses $m_{s}$ and scanning rates *β* requires a normalisation of the measured heat flow signal. For this reason, the specific heat capacity $c_{p_{\mathrm{excess}}}$ [25] is calculated according to:

(1) $$c_{p_{\mathrm{excess}}}=\frac{{\dot{Q}}_{s}-{\dot{Q}}_{\mathrm{BL}}}{m_{s}\beta }\;\left(\mathrm{in}\;J\cdot g^{-1}\cdot K^{-1}\right)$$

with heat flow of baseline ${\dot{Q}}_{\mathrm{BL}}$ and sample measurement ${\dot{Q}}_{s}$.

Remaining artefacts such as overshoots at the start and end of a scanning step were removed. The residual curvature of $c_{p_{\mathrm{excess}}}$-curves can be compensated for with a polynomial fit. This was applied only for heating curves with scanning rates of 0.01 K s^−1^ and 0.03 K s^−1^, because, for this data processing step, reaction free zones at low and high temperatures are necessary [4,21]. 

The slow heating experiments (0.01–0.1 K s^−1^) consist of 4–6 sample measurements and 2–3 baselines. In the heating rate range of 0.3–5 K s^−1^, eight sample measurements and four baselines were performed for each scanning rate. For indirect DSC, four sample measurements and two baselines for each maximum temperature and each heating rate were conducted. The average curves of these experiments are plotted in the diagrams. In total, more than 220 DSC experiments were performed.

### 2.4. Thermo-Mechanical Analysis and Hardness Testing

**Figure 3:**
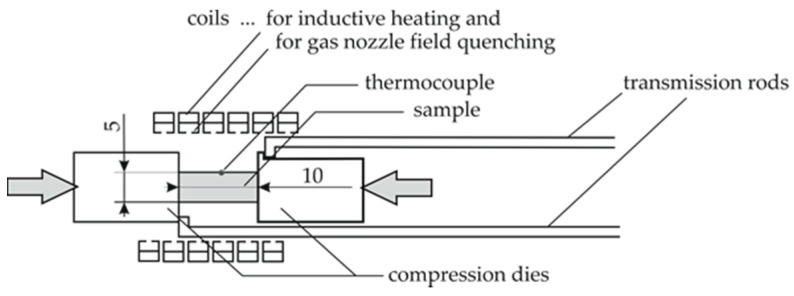
Schematic of cylindrical compression sample inside quenching and deformation dilatometer type Bähr 805 A/D.

Thermo-mechanical analysis measures the deformation of a material under compression or tension as a function of temperature. To analyse the mechanical properties of the aluminium alloy 6082 T651 depending on the parameters of a thermal welding cycle, a thermo-mechanical analysis has been performed in the quenching and deformation dilatometer type Bähr 805 A/D. A schematic of the cylindrical compression sample inside the testing machine is shown in Figure 3. During the investigation, the specimens with geometrical dimensions of Ø 5 mm × 10 mm are heated inductively by the surrounding induction coil. An additional perforated inner coil was used for inert gas cooling. The temperature of the specimen was controlled with thermocouples spot-welded onto the specimen surface. The samples retrace the temperature–time profiles, which were measured in the HAZ during welding. The compression tests were carried out after seven days natural aging at about 20 °C with a deformation rate of 1 mm/s. Thereby, force–displacement curves were recorded. Every combination of heating rate and temperature was repeated three or four times and revealed a good reproducibility. The determined load-displacement diagrams were evaluated to flow curves representing true stresses and true strains.

During the evaluation of the compression tests, the absolute values of forces and displacements are calculated so that only positive strains and stresses are shown in the diagrams. These data can be compared directly with the results of the tensile tests.

For the hardness curve over the cross-section of weld seams, hardness values (HV1) were ascertained with the micro hardness tester HMV-2 from Shimadzu, Kyoto, Japan.

### 2.5. Tensile Tests on Welded Joints

To obtain the mechanical properties of the fusion zone (FZ), tensile tests on round specimens were conducted. The specimens had a diameter of 6 mm and were machined out of a V-shaped butt weld. Due to manufacturing limitations this specimen contained not only the weld material, but also small parts of the heat affected zone. Therefore, the results of these tests must be seen as integral values of the fusion zone and adjacent heat affected zone material. The displacements were measured by an extensometer.

**Figure 4:**
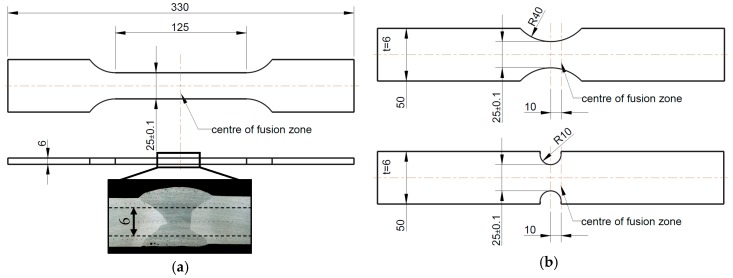
Geometries of the (**a**) smooth and (**b**) notched butt welded flat specimen for investigation of the HAZ, lengths in mm.

To determine the mechanical behaviour of the heat affected zone, tensile tests on whole welded joints were conducted. Two plates of the base material were joined with an X-shaped butt weld, as described in Chapter 2.2. To obtain flat specimens the 10 mm thick welded plates were milled to 6 mm thickness (Figure 4a). Smooth and notched specimens (notch radius of 10 mm and 40 mm, Figure 4b) with a width of 25 mm in the smallest cross section were manufactured. Displacements and strains on the surface of the flat specimens were measured with a 2D digital image correlation system. Therefore, the surface of the specimens was prepared with a speckle pattern. The camera resolved the surface with a pixel size of 0.03 mm. The majority of the speckles had a size of 2 × 2 to 4 × 4 pixels. The data was processed with the software VIC 2D 6 (Correlated Solutions, Irmo, SC, USA).

The DIC offers the possibility to place several virtual extensometers on the specimen surface with a freely chosen length. Therewith, force–displacement curves of several zones of the specimen can be obtained. The dimensions of the zones were first derived from hardness measurements and then compared with the DSC and TMA experiments.

For this investigation, the specimen was divided into four areas of interest: fusion zone and three areas in the heat affected zone (Z1, Z2 and Z3). Z3 was chosen such that differences with the base material (BM) are small and therefore the properties of the BM can be assumed (see hardness measurements in Chapter 3.3). Necking and fracture of the specimen was expected in Z1. Z2 filled the area between Z1 and Z3.

With the virtual extensometers, the force–displacement curves of each individual material zone were measured along with a global force–displacement curve including all zones. For the global curve, the virtual extensometer had a base length of 65 mm, whereas the extensometers of Z1 and Z2 were applied over the whole zone length of 12 mm and 9 mm, respectively. Accurate strain measurements can be achieved with the virtual extensometers due to their undeformed length of at least 300 pixels. Material properties from Z1 and Z2 can be obtained with this method without manufacturing separate specimens.

### 2.6. Determination of True Stress–Strain Curves

Tensile tests can be evaluated to determine the flow curve of a material. As long as uniform elongation occurs in the tests, the flow curve (equivalent von Mises strain $\sigma_{\mathrm{vM}}$ over total equivalent plastic strain $\varepsilon_{\mathrm{pl}}$) can be calculated analytically as follows. First, the true strain *ε*

(2) $$\varepsilon \;=\;\ln(1\;+\;\varepsilon_{e})$$

and true stress *σ*

(3) $$\varepsilon \;=\;\sigma_{e}\left(1\;+\;\varepsilon_{e}\right)$$

can be calculated from the engineering stresses $\sigma_{e}$ and engineering strains $\varepsilon_{e}$. In this case, the true stress equals the von Mises equivalent stress $\sigma_{\mathrm{vM}}$. The plastic strain $\varepsilon_{\mathrm{pl}}$ can be calculated by

(4) $$\varepsilon_{\mathrm{pl}}\;=\;\varepsilon \;-\frac{\sigma }{E}\;$$

After onset of necking of the specimen, the stress state is not uniaxial anymore. To obtain the flow curves beyond the onset of necking, there are several analytical approaches. One often used method is to fit the values obtained by Equations (3) and (4) with a simple power law of the form

(5) $$\sigma_{\mathrm{vM}}{{\;=\;K\varepsilon }_{\mathrm{pl}}}^{n}\;$$

Another possibility is to calculate the parameters *K* and *n* of Equation (5) with the true stresses $\sigma_{m}$ and plastic strains $\varepsilon_{m}$ at the beginning of necking. The power law becomes

(6) $$\sigma_{\mathrm{vM}}{\;=\;\sigma }_{m}{\left(\frac{\varepsilon_{\mathrm{pl}}}{\varepsilon_{m}}\right)}^{\varepsilon_{m}}\;\mathrm{for}\;\varepsilon_{\mathrm{pl}}\;\geq {\;\varepsilon }_{m}\;$$

and allows an extrapolation of the experimental data beyond the onset of necking.

However, neither method considers experimental results after the start of necking. Therefore, numerical simulations were conducted with the finite element program MarcMentat2013 to obtain flow curves with an iterative procedure. On the one hand, round specimens were simulated with rotational symmetric half models. On the other hand, 3D volume models were used to simulate flat specimens. In contrast to the geometry of the specimens, the deformation of the welded specimens is not symmetric in the tension direction due to strain localisation in the HAZ at one side of the fusion zone. Therefore, a quarter model with symmetry in width and thickness directions was used.

In this iterative procedure, the flow curve of the material is changed in a way that the resultant force–displacement curve in the simulation equals the force–displacement curve of the experiment. The detailed procedure was described by Gannon [25].

This method can be used for the base material and fusion zone material, since specimens with homogenous behaviour are assumed. For the HAZ, this method is not useable without modification, because the flat specimens do not consist of a homogeneous material (see Figure 4a). Furthermore, no necking or failure occurs in the Z2. Accordingly, the experimental stress–strain curve of the Z2 does not reach the tensile strength for this zone and $\sigma_{m}$ and $\varepsilon_{m}$ are unknown. Thus, the experimental result for the flow curve of the Z2 is extrapolated with a fitted power law given in Equation (5).

The flow curve of the Z1 can be obtained by iteration, but instead of using one single material the whole specimen with FZ, Z1, Z2 and Z3 (assumed properties of the BM) and their respective flow curves was modelled. The simulation of a complete specimen ensures that the edges of the Z1 behave correctly, because the different strengths of the adjacent Z2 and fusion zone hinder the deformation in width direction.

## 3. Results and Discussion

### 3.1. Temperature–Time Course in Heat Affected Zone

**Figure 5:**
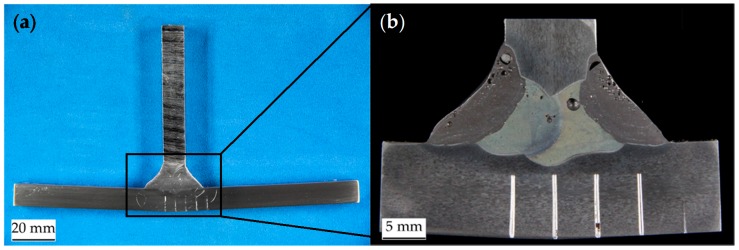
(**a**) Cross-section of welded T-joint; and (**b**) macro image of bores for thermocouples.

The cross section of the welded joint, which was used for temperature measurements, is shown in Figure 5 including thermocouple bores. The thermocouple wires were located at the end of the blind holes, so the distance between each weld bead and the points of temperature measurement was determined with these cross-section images.

**Figure 6:**
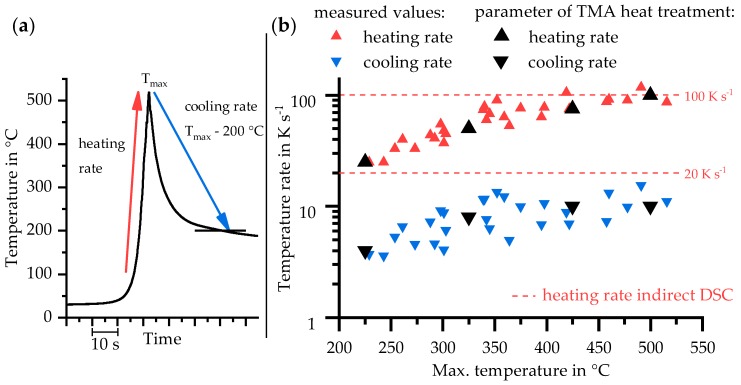
(**a**) Typical measured temperature–time course in HAZ; and (**b**) heating and cooling rates in the HAZ during MIG welding of EN AW-6082 depending on the maximum temperature and the resulting parameter of TMA heat treatment as well as the heating rates for indirect DSC.

A typical temperature–time course in HAZ during welding and its three analysed parameters (heating rate, T_max_, and cooling rate) are shown in Figure 6a. The heating in all recorded courses was nearly linear over a wide temperature range. The maximum temperature (T_max_) was reached without a holding time and the cooling started immediately with a Newtonian course. Below 200 °C, the temperature decreases very slowly due to the relative small dimensions of joined plates, which heated up significantly. Therefore, only the cooling between T_max_ and 200 °C was used to calculate the mean cooling rate.

The analysed heating and cooling rates in the HAZ during welding are plotted against T_max_ in Figure 6b. In principle, the heating and cooling rate increase as the maximum temperature rises, although a scattering of measured values occurs.

The three analysed parameters of temperature measurement revealed:

- Linear heating rates: 25–118 K s^−1^
- Maximum temperatures (T_max_): 229–516 °C
- Averaged cooling rates between T_max_ and 200 °C: 3.5–15 K s^−1^.

**Figure 7:**
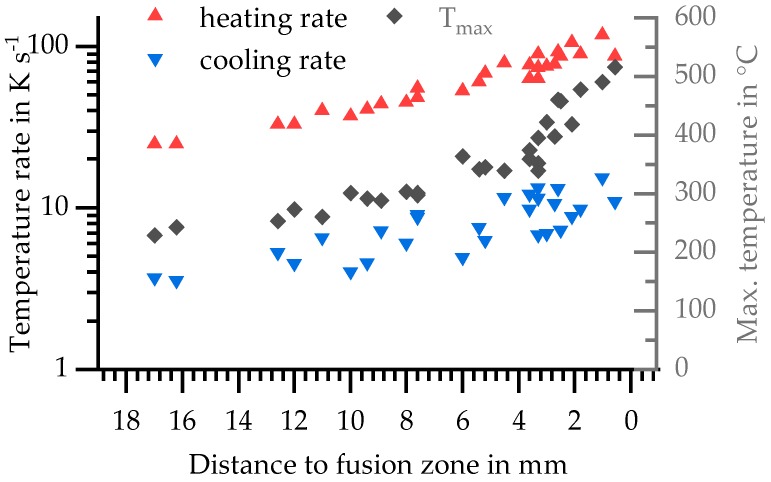
Parameters of temperature–time course dependent on distance to the weld seam.

Because the maximum temperature correlates with distance from the fusion zone, these results are also plotted against the distance to weld bead in Figure 7.

These results, temperature rates and corresponding maximum temperatures, retrace different positions in the HAZ and were selected as parameters for TMA in this study. They are marked with black symbols in Figure 6b and given in Table 5. The chosen heating rates of indirect DSC (20 K s^−1^ and 100 K s^−1^) are in the minimum and maximum range of these values.

### 3.2. Precipitation and Dissolution Behaviour of EN AW-6082 T651 in a Wide Dynamic Range

**Figure 8:**
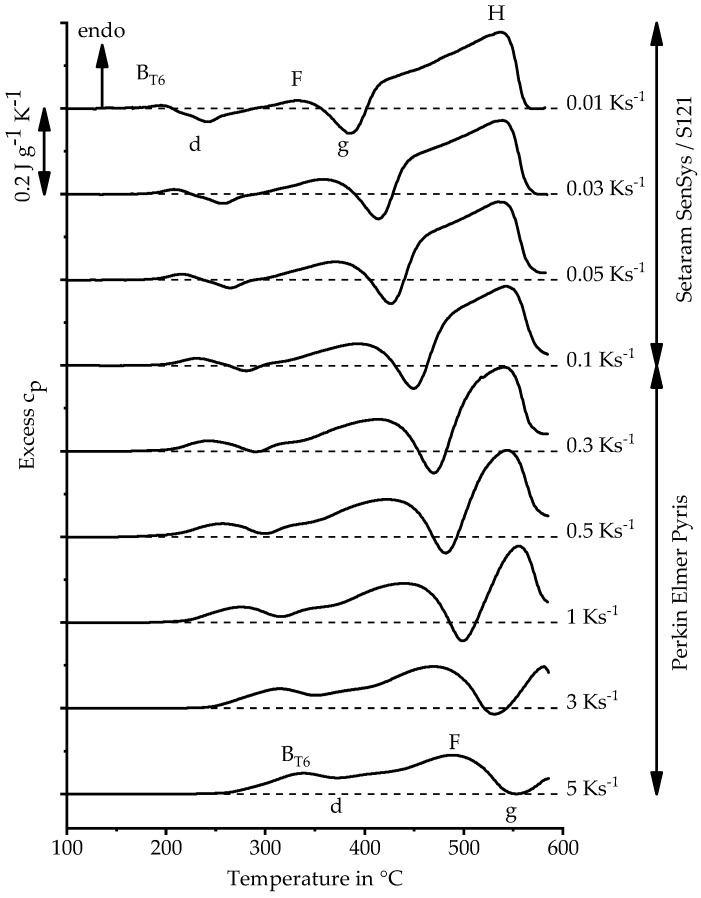
Direct DSC heating curves of EN AW-6082 T651 heating rates 0.01 K s^−1^ to 5 K s^−1^.

The excess heat capacity curves of heating the alloy EN AW-6082 with initial state T651 over a heating rate range from 0.01 K s^−1^ to 5 K s^−1^ up to 585 °C are plotted in Figure 8. During heating of aluminium alloys, dissolution and precipitation reactions occur. Precipitations were measured as exothermic peaks and dissolution as endothermic peaks. These reactions are alternating and overlap each other. Thus, the DSC curves show only the resulting sum signal, and only the initial temperature of the first and the final temperature of the last reaction are true signals.

The DSC curve recorded by Osten et al. [21] with another batch of EN AW-6082 with a 0.01 K s^−1^ heating rate resembles the curve from this study with the same scanning rate. There are only slight differences in reaction behaviour at slow scanning rates, which can be explained by differences in chemical composition, but the sequence of reactions is the same. Therefore, their interpretation of the reaction sequence is used in this study. The reactions were labelled here with the same characters [21].

The first peak B for the initial state T6 is induced by the dissolutions of GP-zones and β″, with β″ being the phase which effects the maximum strengths of Al-Mg-Si alloys [13]. The peak d corresponds to either the precipitation of β″ or β′ depending on initial state [13,15]. For the initial state T651, there is probably only a precipitation of β′, because β″ is already dissolved in the previous reaction. The reactions which cause the peaks F and g belong to the dissolution of β′ and the precipitation of β (Mg_2_Si). The dissolution of the remaining precipitations, especially β (Mg_2_Si), is recorded as final peak H. At very slow heating rates, there is a reaction-free range following peak H, which indicates a complete dissolution of these particles [21].

As the heating rates increase, there is a shift of reactions to higher temperatures, which also results in an incomplete dissolution with fast heating. Furthermore, the curves shift in the endothermic direction. However, it is unlikely that dissolution will increase at faster heating rates. Rather, it can be assumed that the shift is thus caused because precipitation reactions are significantly more suppressed than dissolution reactions.

**Figure 9:**
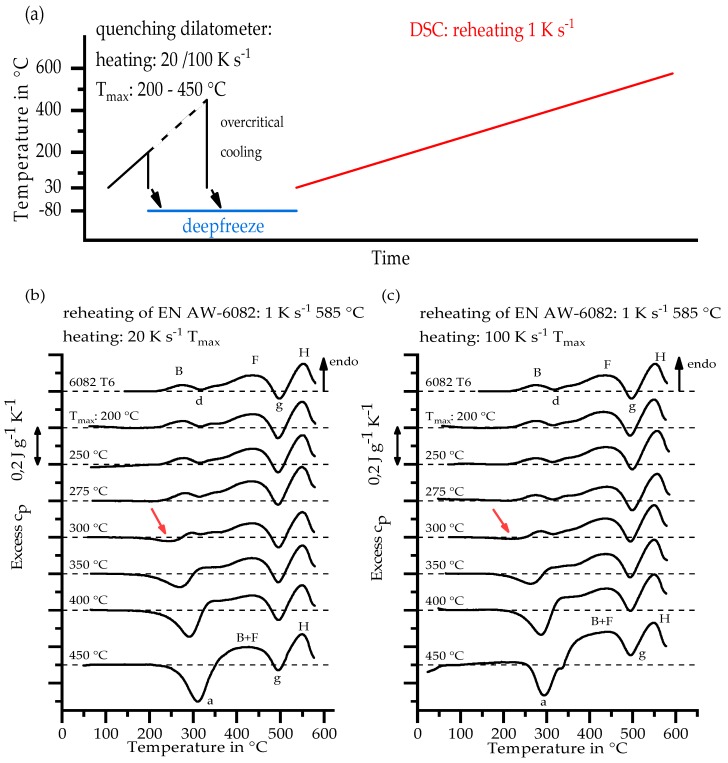
Indirect DSC: (**a**) schematic temperature–time course; and reheating DSC curves of heating rates: (**b**) 20 K s^−1^; and (**c**) 100 K s^−1^.

Figure 9a displays the temperature–time course of the indirect DSC method. To maintain the condition at T_max_ and to prevent quench-induced precipitation, quenching is performed with maximum gas flow after the first heating. The average cooling rates *β* of the Newtonian cooling course, which depended on the temperature interval considered, are listed in Table 6.

Fröck et al. [24] used the same batch of 6082 to investigate the influence of different solution conditions on the precipitation behaviour during subsequent cooling. For an incomplete solution state (after 540 °C for 1 min), the upper critical cooling rate (uCCR) of 100 K s^−1^ was ascertained. The cooling rates of the heat treatment for indirect DSC are higher than this uCCR in temperature ranges above 100 °C. It can thus be assumed that no significant precipitation reactions took place during cooling and the state of the material reached at maximum temperature remains.

The reheating curves are shown in Figure 9b,c. The reaction peaks are given the same characters as in Figure 8. Low curvature is present in the curves, which can give reasons for slight quantitative differences between single curves. This is particularly apparent at higher temperatures, e.g., the peaks g and H, or the slope of reaction free zone are influenced by this remaining curvature. Nevertheless, the development of reactions is clearly visible. The reheating curves of the investigated heating rates 20 K s^−1^ and 100 K s^−1^ show no significant differences for the same T_max_. Depending on T_max_, there is a substantial development in the reheating curves for each heating rate. In conclusion, the reactions taking place in the HAZ are mainly dependent on T_max_ and are less dependent on the heating rate, at least in the investigated range.

The reheating curves from the initial state EN AW-6082 T651 to T_max_ of 275 °C are almost identical. That means no significant reactions take place until heating to this temperature. From T_max_ 300 °C an exothermic reaction starts (see arrows in Figure 9b,c). These reaction peaks increase with a higher maximum temperature of first heating. During the first heating, existing precipitates are dissolved increasingly with rising temperature. A supersaturation occurs due to overcritical cooling, which causes the measured precipitation reactions during reheating. This dissolution reaction B_T651_ during rapid heating is crucial for softening in the HAZ.

**Figure 10:**
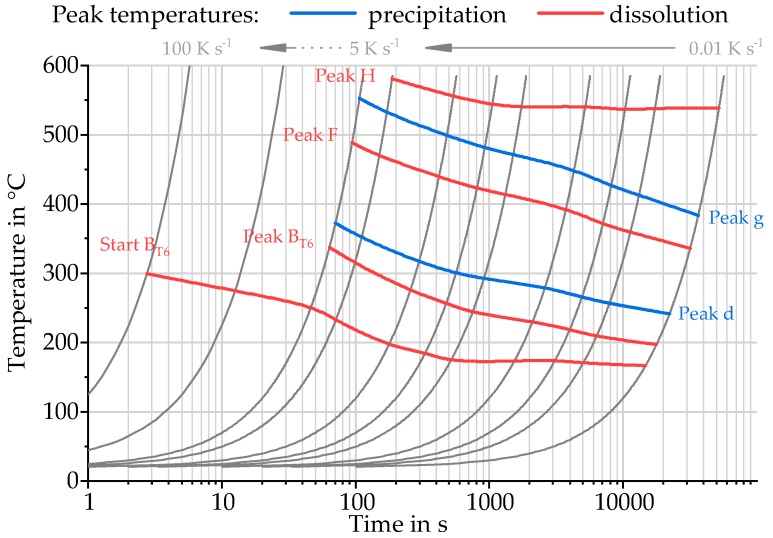
Continuous heating dissolution diagram EN AW-6082 T651 heating rates 0.01 K s^−1^ to 100 K s^−1^.

The reaction peaks determined by direct DSC and the dissolution reaction B_T651_ determined by indirect DSC are plotted in temperature–time courses of investigated heating experiments, to create a continuous heating dissolution diagram for a wide range of heating rates, as shown in Figure 10.

The temperatures of dissolution or precipitation reactions during heating of EN AW-6082 T651 within a range of 0.01 K s^−1^ to 100 K s^−1^ can be taken from this diagram. For heating of 20 K s^−1^ to 100 K s^−1^, investigated with indirect DSC, only the start of the dissolution reaction B_T651_ can be determined at temperatures between 275 °C and 300 °C.

### 3.3. Mechanical Properties of the HAZ

**Figure 11:**
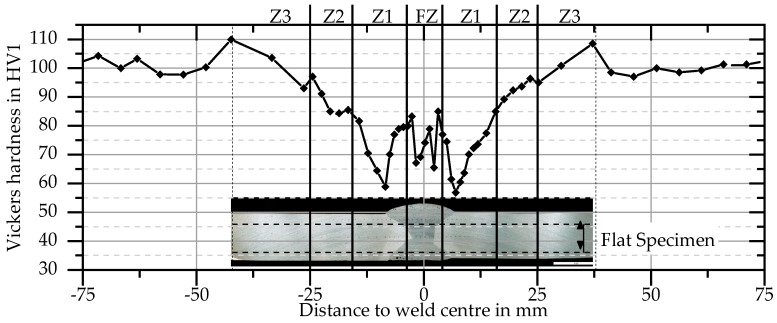
Hardness after welding and natural aging in plate centre.

The results of hardness tests in Figure 11 provide an overview of properties as a function of distance to the weld centre. At a distance from the weld centre of more than 50 mm a constant hardness of about 100 HV1 was measured in the base material 6082 T651. At about 40 mm, a maximum hardness of 110 HV1 is reached. One reason for the increase in hardness may be that the initial state T651 was slightly underaged and the welding heat causes artificial ageing at this point. With decreasing distance, the hardness decreases significantly to a minimum of about 60 HV1. The hardness increases in the direct vicinity of the FZ. Hardness of the FZ was about 70–80 HV1.

**Figure 12:**
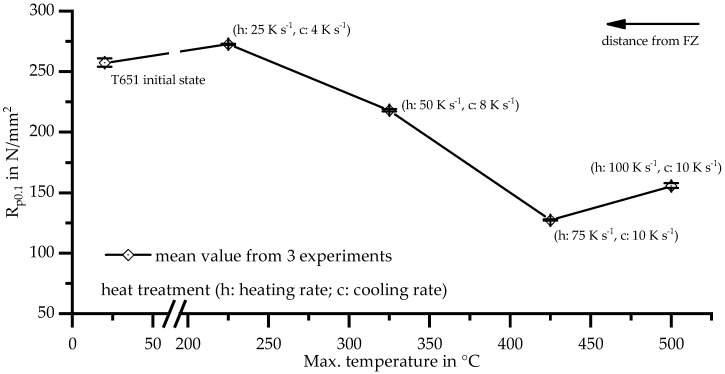
Yield strength after welding cycle and seven days natural aging depending on maximum temperature of short term heat treatment.

In Figure 12, the results of TMA with parameters according to Table 5 are plotted against T_max_ for the short term heat treatment. The yield strength has been measured after seven days of natural ageing. Compared with the initial state, there is a small increase for T_max_ 225 °C. From T_max_ 225 °C to 425 °C, the yield strength decreases by about half to less than 130 N/mm^2^. For the highest investigated T_max_ of 500 °C the yield strength increases slightly.

Microstructure analyses (SEM and TEM) were performed by Fröck et al. [24] with the same material after annealing at different maximum temperatures. During annealing, both complete and incomplete dissolution of secondary phase particles was achieved depending on the maximum temperature. As Figure 9 shows, there will be an incomplete dissolution for fast heating rates. In consideration of the quasibinary phase diagram Al-Mg_2_Si [15], the same phases are expected after the TMA welding heat treatments as after solution annealing at 540 °C [24].

Because maximum temperature correlates with distance to the FZ, the course of the yield strength (Figure 12) depending on maximum temperature is similar to the hardness profile (Figure 11).

Regarding DSC and TMA, the HAZ of 6082 T6 can be divided in four areas.

A. Above 425 °C, solution annealing takes place. Rapid quenching near the FZ causes a supersaturated solid solution with potential for age hardening. Yield strength increases again after natural aging.
B. From 275 °C to 425 °C, β″ precipitates increasingly dissolve and yield strength decreases.
C. Weak precipitation of β″ happens at a temperature range of 225 °C, which leads to a slight increase in hardness and strength, but is hardly detected with DSC.
D. At a distance of more than 50 mm (below a certain T_max_), the T6 state consisting of β″ precipitates remains nearly unchanged. Hardness is not affected.

### 3.4. Flow Curves in a Welded Joint

For the calculation of the flow curve of the base material and the fusion zone the engineering stress–strain curves determined from tensile tests on separate round specimens have been used. The mechanical properties of the fusion zone material were also determined from these tensile tests and are presented in Table 7. The chemical composition of the FZ according Table 1 appears in the range of cast aluminium alloys, which also roughly applies for its mechanical properties.

**Figure 13:**
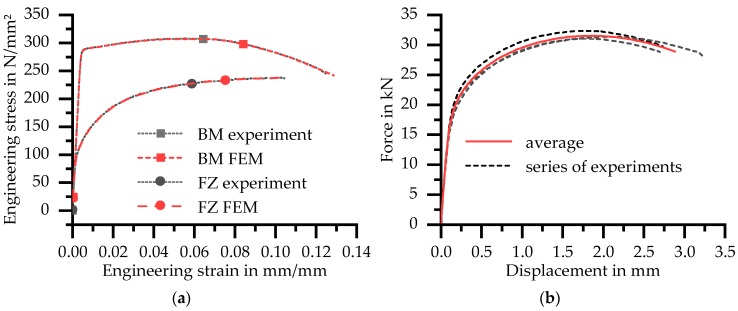
(**a**) Comparison between measured and calculated engineering stress–strain curves of base material and weld material; and (**b**) force–displacement curves of butt welded flat specimen.

Whereas the base material shows ductile failure with necking after reaching the ultimate tensile strength, the fusion zone material fails without any noticeable necking (see Figure 13a). Therefore, the combined analytical and numerical approach described in Chapter 2.6 was used to calculate the flow curve of the base material. Numerical iterations were not necessary for the fusion zone material, since no necking and therefore no multiaxial stress state was present. The flow curve of the fusion zone was simply calculated by Equations (2)–(4). An extrapolation with Equation (6) extends the curve to a larger range of strains. To validate the obtained flow curves, a comparison between calculated and measured technical stress–strain curves is also shown in Figure 13a. No differences between the measured and simulated curves are visible.

Whereas all tests with the base and fusion zone material showed very good repeatability, the global force–displacement curves of the three tested welded flat specimens showed slight differences (see Figure 13b). It is assumed that the differences occur because of irregularities in the weld seam in length direction as well as due to specimen manufacturing from slightly different areas over the sheet thickness. To overcome the differences between curves, one average curve was used for comparison reasons with numerical simulations.

In addition to the global force–displacement curve, local force–displacement curves for the zones Z1 and Z2 were also determined by using the DIC. The respective lengths and positions of the material zones were derived from hardness measurements as shown in Figure 11. Z1 is the area between 4 mm and 16 mm distance to the centre of the fusion zone. This is the area in which fracture occurs during tensile tests. Z2 ends at 25 mm distance to the centre of the fusion zone when the hardness values increase to about 95% of the base material (i.e., about 95 HV1). For distances to the fusion zone larger than 25 mm (Z3), the properties of the unaffected base material are nearly reached.

**Figure 14:**
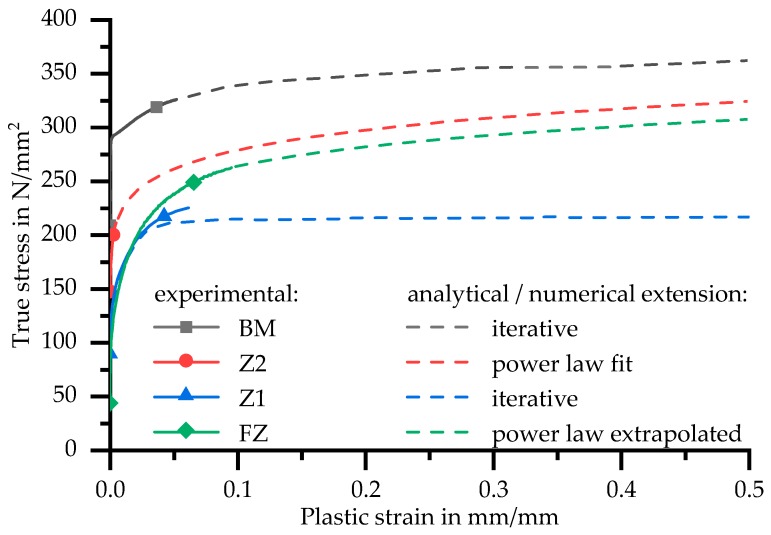
Flow curves of base and weld material, Z1 and Z2.

For this arrangement, the experimental force–displacement data for Z2 only allows a calculation of the flow curve until about 0.3% plastic strain, because failure and strain localisation occurred in Z1. The curve of Z2 is extended to higher strains by fitting a power law according to Equation (5). The flow curve of Z1 is obtained afterwards through iteration with numerical simulations. In contrast to the base material, it was not possible to use Equations (2)–(4) until necking occurs (see Figure 14).

Due to the inhomogeneity of the HAZ, uniform elongation cannot be assumed until the maximum force is reached. Therefore, the experimental data were used as initial values for the numeric iteration only as long as agreement was maintained between the measured and calculated force–displacement curves.

### 3.5. Validation of Obtained Flow Curves in the HAZ

**Figure 15:**
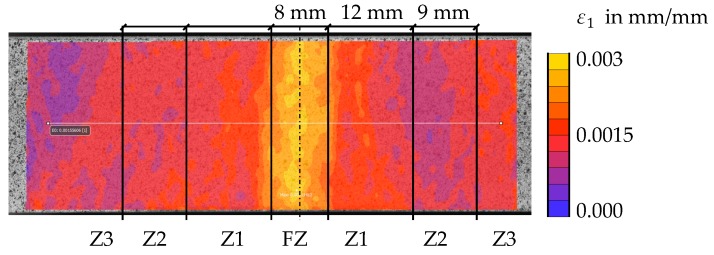
Strain distribution in a welded flat specimen at low global displacements (0.1 mm).

The results of the tensile tests with butt welded flat specimen are here described in more detail. To validate the calculated curves, the strain distribution in the experiment (DIC) can be compared with the numerical results. Therefore, the maximum principal strain $\varepsilon_{1}$ was calculated in the DIC software at the specimen’s surface. First, Figure 15 shows that no uniform elongation of the specimen is present even at low global displacements (maximum strain of 0.3%). Whereas the hardness measurements (see Figure 11) suggest the highest strain in Z1 next to the fusion zone, the fusion zone material dominates the deformation of the specimen at low strains. The behaviour of the flow curves (Figure 14) of the two zones explains this phenomenon: at low strains, the flow stress of the fusion zone material is less than the flow stress of Z1. A certain amount of strain hardening needs to occur for Z1 to dominate the deformation behaviour of the specimen.

**Figure 16:**
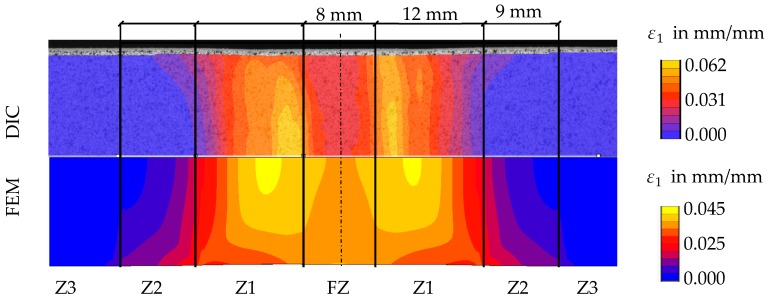
Comparison of the strain distribution in a butt welded flat tensile specimen at 1.3 mm global displacement: in the experiment (**top**); and in the FE simulation (**bottom**).

The top of Figure 16 shows the measured strain distribution of the specimen at 1.3 mm global displacement. In contrast to the strain distribution at low displacements, here, the highest strains occur almost symmetrically next to the fusion zone in Z1. For comparison, the bottom of Figure 16 shows the maximum principal strains calculated by the finite element (FE) simulation at the same displacement.

At first glance, the strain distribution shows good agreement between model and experiment. In both cases, the maximum strain is located in Z1. Whereas there are still noticeable strains in the fusion zone, the strain decreases within a few millimetres in Z2 to almost negligible strains in Z3. Since Z3 and Z2 deform less than Z1, the deformation of Z1 is constrained in the width direction. This constraint causes higher strains in Z2 at the edge of the specimen than in the middle. The constraining effect on the different material deformations becomes stronger in the simulation than in the experiment, because the FE model has no continuous change in material properties but rather an explicit change at the end of each material zone.

**Figure 17:**
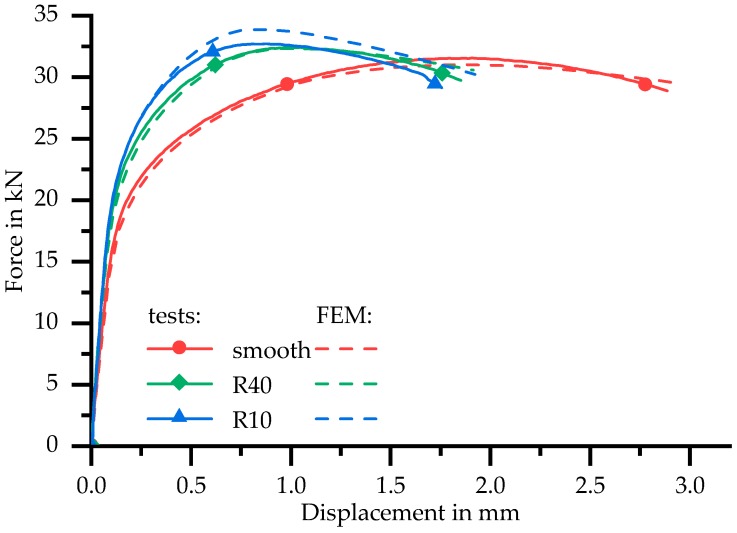
Force–displacement curves of notched and smooth butt welded flat specimen.

Another difference becomes visible by comparing the maximum strain values. The measured maximum strain is higher than in the numerical simulation and located closer to the fusion zone. It has to be pointed out that differences in maximum strain occur even though the measured and simulated force–displacement curves of the whole specimen are almost identical (see Figure 17). This is possible because the flow curve of Z1 averages a quite large area of the HAZ compared to high changes in hardness and the presumed mechanical properties in this zone. Since for example the lowest yield stress is averaged to a higher value, a smaller strain peak will be calculated.

To investigate the behaviour of the HAZ in different multiaxial stress states and to validate the obtained flow curves in more detail, tensile tests and numerical simulations of notched specimens were conducted. Figure 17 shows a comparison of three different specimen shapes: smooth, large notch radius (40 mm) and small notch radius (10 mm) with equal nominal cross sections.

As it is well known, a notch will increase the maximum force: the smaller the notch radius, the higher the maximum force. The experimental results confirm this fact. However, the increase of the maximum force is small. This indicates that the inhomogeneity of the material dominates over the geometric effect due to the notch. The increase of maximum force is calculated by the FE simulations as well. However, the simulated and measured force–displacement curves of the specimen with large notch radii have good agreement, while the simulation overestimates the maximum force of the sharp notched specimen. Due to the material properties averaged in Z1, expressed by the flow curve, a larger force is required in the finite element simulation in order to map the local strain concentration in the notch root.

### 3.6. Correlation between Results of Tensile Tests and TMA of HAZ

When comparing the results of different methods, the type of joint used for temperature measurement (T-joint) and tensile test specimen (butt joint) must be considered. Whereas in a butt weld the heat can only be dissipated in two directions, the T-joints consists of three segments. Higher T_max_ as a function of distance and lower cooling rates can therefore be expected on the butt weld.

**Figure 18:**
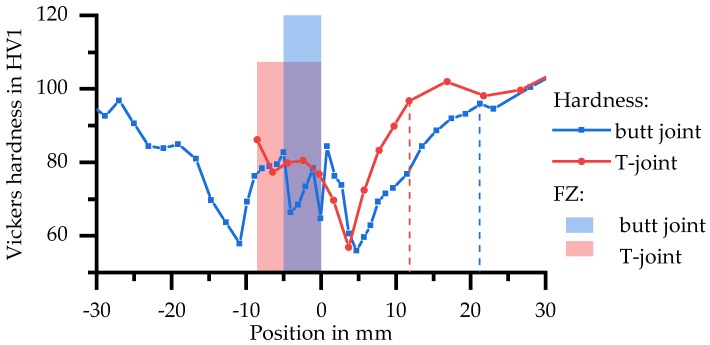
Hardness development in butt and T-joint depending on the position.

Hardness profiles (see Figure 18) of both welds were recorded in order to compare the welds and in particular the size of the HAZs with each other.

For the T-joint, the values of the FZ and the vertical plate are shown. In HAZ the hardness first decreases to a minimum, which is at a distance to FZ of 4 mm in the T-joint and at 5–6 mm in the butt joint. In further course the hardness increases until the initial value of about 100 HV1 is reached at 12 mm (T-joint) and 21 mm distance (butt joint) respectively. The courses of hardness are the same and the locations of, e.g., the minimum or initial hardness, match, considering the geometrically changed distribution of T_max_.

**Figure 19:**
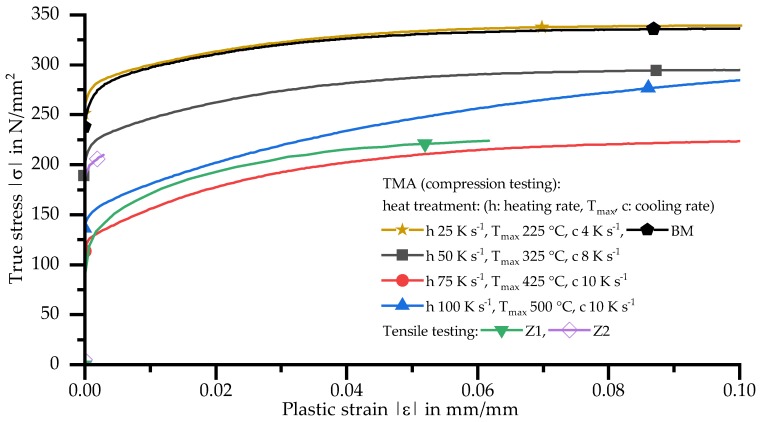
Flow curves of real and retraced HAZ determined with tensile and compression tests. The compression tests are carried out after the welding cycle and seven days of natural ageing.

Flow curves of the HAZ determined with tensile test and TMA can therefore be compared as seen in Figure 19.

The curve of Z1 is enveloped by the retraced HAZ with T_max_ 500 °C and 425 °C for low plastic strains. The experimentally determined flow curve of Z2 is only available up to the maximum stress of Z1, but it can be assumed that it follows the curve with T_max_ 325 °C. These results are in good agreement regardless of the different heat input in tensile and TMA samples. Whereas the tensile tests were carried out with specimens heated three times up to different maximum temperatures (three weld beads), the TMA specimens were subjected to a single, precisely defined short-term heat treatment.

The lowest mechanical properties are obtained by TMA with T_max_ 425 °C. By evaluating tensile tests in Z1 with the combined numerical and analytical approach described previously, the low mechanical properties as in the TMA cannot be determined due to the averaging in Z1. Averaging over the area of Z2 leads to a curve similar to the TMA flow curve with T_max_ 325 °C, even though no significant difference to the BM is expected at the end of Z2. As Figure 12 shows, the short-term heat treatment with T_max_ 225 °C results in maximum strength just above that of the BM.

It becomes visible that a combined approach with tensile tests, DIC and numerical simulations and DSC and TMA following temperature profiles during welding can lead to an improved description of material behaviour in different areas of a weld for a specific welding process and geometry. With knowledge of the maximum temperatures depending on the distance to the FZ, phase transformations obtained by DSC and material properties obtained in a TMA can therefore deepen understanding of the microstructural changes in the HAZ and refine numerical structure–mechanical simulations of welded components.

## 4. Conclusions

In this study, HAZ properties of welded joints made of AlMgSi wrought alloy EN AW-6082 T651 were investigated using several combined methods. The following conclusions can be drawn from the consideration of the individual results and their mutual discussion:

1. Dissolution and precipitation reactions in different areas of the HAZ can be analysed in situ with DSC. To retrace the thermal history in the HAZ, the temperature rate range was extended with the indirect DSC method.
2. There is a good agreement between results of phase transformations determined with DSC and changes in mechanical properties measured with TMA.
3. The softening in HAZ is strongly dependent on peak temperature. With increasing peak temperature, the initial state is increasingly dissolved and the material is softened to a minimum. Near the FZ, the mechanical properties increase due to strong dissolution of alloying elements and the associated potential for natural aging.
4. The development of dissolution and precipitation can be described by continuous heating dissolution diagrams, similar to welding-transformation diagrams of steels.
5. Mechanical properties from TMA and results of tensile tests on welded joints show good agreement in relevant HAZ zones.
6. Flow curves of the base material, fusion zone material and two areas in the HAZ in a butt welded joint can be calculated with a combined numerical and analytical approach. DIC measurements can provide the necessary force–displacement data in the HAZ without manufacturing separate specimens.
7. A certain amount of strain hardening in the FZ needs to occur before the HAZ dominates the deformation of welded flat specimen.
8. Small increases of maximum force with decreasing notch radii indicate that the inhomogeneity of the HAZ dominates over the geometric effect due to the notch.
9. Numerical simulations of notched tensile specimens with these flow curves lead to accurate force–displacement curves for large notch radii. The maximum principal strain is underestimated by numerical simulations, because of the averaging of material behaviour in the HAZ.

## Figures and Tables

**Table 1:** Mass fraction of alloying elements in the investigated EN AW-6082 alloy, fusion zone material of a butt joint and weld filler material EN AW-4047, in percent.

Material/alloy	Source	Si	Fe	Cu	Mn	Mg	Cr	Zn
EN AW-6082	OES	0.83	0.38	0.06	0.48	0.92	0.03	0.01
EN AW-6082	DIN EN 573-3	0.7–1.3	≤0.5	≤0.1	0.4–1.0	0.6–1.2	≤0.25	≤0.2
EN AW-4047A	DIN EN 573-3	11-13	0.6	0.3	0.15	0.1	-	≤0.2
Fusion zone material	OES	7.23	0.29	0.03	0.19	0.39	0.02	<0.01

**Table 2:** Mechanical Properties of EN AW-6082 T651 depending on the rolling direction.

Rolling Direction	*E* (N/mm²)	$R_{m}\;(N/\mathrm{mm}\text{²})$	$R_{p0.2}\;(N/\mathrm{mm}\text{²})$	$A_{5}\;(\text{\%})$
0°	70800	308	289	12.2
45°	70000	303	278	13.0
90°	71300	308	284	11.5
Max. Difference	1.8%	1.5%	3.8%	11.8%
DIN EN 485-2 [11]	70000	300	255	9

**Table 3:** Overview on used samples.

Method	Previous Treatment	Geometry	Dimensions in mm
Temperature measurement	Initial state	T-joint *	240 × 160 × 10 plus 240 × 71 × 10
DSC, heat flow	Initial state	Cylindrical	Ø6 × 21.65
DSC, power compensated	Initial state	Cylindrical	Ø6.4 × 1
TMA	Initial state	Cylindrical	Ø5 × 10
Tensile tests	Initial state	Cylindrical **	Ø8 × 48
Tensile tests	Butt welded	Cylindrical **	Ø6 × 36
Tensile tests, DIC	Butt welded	Flat specimen **	25 × 6 (B×T), smooth, R40, R10

* see Figure 2, ** see Figure 4.

**Table 4:** Welding parameters of EN AW-6082 plates.

Joint	Welding Bead	Current (A)	Voltage (V)	Wire Feed (m/min)	Wire Diameter (mm)
Butt joint	1	145	23.5	7.5	1.2
	2 & 3	145	23.5	7.5	1.2
T-joint	1 & 2	204	24.4	9.5	1.2
	3 & 4	188	23.7	8.5	1.2

**Table 5:** TMA parameters retracing HAZ.

Distance to Fusion Zone	Max. Temperature in °C	Heating Rate in K s^−1^	Cooling Rate in K s^−1^
Ca. 2 mm	500	100	10
Ca. 4 mm	425	75	10
Ca. 8 mm	325	50	8
Ca. 16 mm	225	25	4

**Table 6:** Average cooling rates of heat treatment for indirect DSC.

Upper Temperature	Lower Temperature	Average Cooling Rate
450 °C	100 °C	~200 K s^−1^
200 °C	100 °C	~120 K s^−1^
100 °C	30 °C	>32 K s^−1^

**Table 7:** Mechanical properties of the fusion zone material.

Material	*E* (N/mm²)	$R_{m}\;(N/\mathrm{mm}\text{²})$	$R_{p0.2}\;(N/\mathrm{mm}\text{²})$	$A_{5}\;(\text{\%})$
FZ	71800	238	114	10
